# Pilot Clinical Trial to Evaluate In Situ Calcium Phosphate Cement Injection for Conservative Surgical Management of Appendicular Osteosarcoma in Dogs

**DOI:** 10.3390/ani14101460

**Published:** 2024-05-14

**Authors:** Céline Molle, Aquilino Villamonte-Chevalier, Julien Carabalona, Aurélia Klajer, Julien Letesson, Guillaume Ragetly, Bertrand Védrine, Juliette Blondiau, Olivier Gauthier

**Affiliations:** 1 TheraVet, 6041 Gosselies, Belgium; aquilino.villamonte.chevalier@thera.vet; 2 Clinique Vétérinaire Olliolis, 83190 Ollioules, France; carabalona@olliolis.com; 3 Clinique Vétérinaire Eiffelvet, 75015 Paris, France; eiffelvet@gmail.com; 4 Clinique Vétérinaire Lameilhé, 81100 Castres, France; julien.letesson@vetocastres.fr; 5 Centre Hospitalier Vétérinaire Frégis, 94250 Gentilly, France; 6 Clinique Vétérinaire SeineVet, 76000 Rouen, France; bvedrine@yahoo.fr; 7 OCRvet, 59041 Lille, France; jblondiau@ocrvet.com; 8 Département de Chirurgie des Animaux de Compagnies, Centre Hospitalier Universitaire Vétérinaire ONIRIS, 44307 Nantes, France; olivier.gauthier@oniris-nantes.fr

**Keywords:** osteosarcoma, dog, calcium phosphate cement, palliative treatment

## Abstract

**Simple Summary:**

Amputation or limb-sparing are the main surgical options for removing the primary tumor in canine appendicular osteosarcoma (OSA). Usually, these surgeries are complemented by adjuvant therapy to help to prevent metastatic dissemination. However, in some cases, these procedures may not be possible due to various factors such as the limitation of the surgical procedure, the dog’s medical condition, or the owner’s refusal. Herein, a study was carried out to assess the safety and efficacy of an alternative surgical approach called cementoplasty, which uses a calcium phosphate bone substitute. Involving 12 dogs, the study focused on evaluating its ability to reduce pain and lower the risk of pathological fractures. The results indicated an enhanced quality of life post-treatment, along with a decrease in the incidence/occurrence of pathological fractures. This study highlights cementoplasty as a safe palliative alternative to conservative surgical methods, offering potential benefits in the overall management of canine osteosarcoma.

**Abstract:**

Cementoplasty is a minimally invasive procedure that consists of injecting a bone substitute into the tumor lesion to provide bone reinforcement and alleviate pain. This study aimed to demonstrate the feasibility, safety, and efficacy of cementoplasty with a calcium phosphate cement in osteosarcoma to reduce pain and preserve limb function. Throughout the 6-month study, dogs received no adjuvant therapy, and dogs’ evaluations included a clinical examination, monitoring of postoperative complications, radiographic follow-up, and assessment of limb function and pain scores. Out of 12 dogs enrolled, 10 were withdrawn before study completion due to deterioration in their general condition. Nine (9) dogs were followed until D28, six until D56, and two until D183. Compared to D0, more than 50% of the dogs showed improvement in both veterinarian and owner scores at their final visit. Throughout the study, 10 major and 4 minor complications were reported, all unrelated to the procedure. This open non-controlled study provides first evidence of the feasibility, safety, and efficacy of cementoplasty procedure using a calcium phosphate bone cement to relieve pain and preserve limb function in dogs suffering from appendicular osteosarcoma.

## 1. Introduction

Appendicular OSA accounts for more than 85% of primary bone tumors affecting dogs and for approximately 5% of all canine neoplasms [1,2,3,4,5]. OSA is characterized by an aggressive and invasive behavior occurring most commonly in the metaphyseal region of forelimb long bones [2,5,6,7]. Several OSA risk factors have been identified. Some dog breeds appear to have a genetic predisposition to OSA with large or giant breeds being almost exclusively exposed to the disease [2,4,5,8,9,10,11]. However, size, body weight, and especially height are considered more important risk factors than breed per se [12,13]. Furthermore, male dogs have been reported to be at risk, with neutering being associated with a potential increase in the risk factor of the disease [5,7,13]. Finally, OSA tends to occur in middle-aged to older dogs (6 to 10 years of age), with some reports showing another incidence peak at 2 years of age [5,7,12,13].

Dogs with appendicular OSA are often presented for acute or chronic onset of weight-bearing or non-weight-bearing lameness. Local limb swelling can also be observed. The mass is usually firm and often painful on palpation [1,5]. OSA results in radiographic evidence of both osteoproductive and osteolytic lesions [13]. Suspected lesions need to be confirmed by histological examination [5,13]. Canine OSA is associated with a high metastatic rate with up to 90% of the dogs having micrometastases, although only 15% have radiographically visible metastases at the time of diagnosis [3]. The lungs are the most commonly affected organs, but metastases have also been detected in regional lymph nodes, distant bone, and soft tissues [3,7,9,13,14]. Chest X-rays and fine needle aspiration of the lymph nodes are especially essential to determine the extent of the disease’s progression. Pathological fracture is a well-recognized complication of canine OSA. Indeed, the deterioration of the viscoelastic properties of the affected bone may result in increased plastic deformation and subsequent fracture. As a result, the fractures usually develop spontaneously or with minimal trauma [6,15]. Micrometastases and lung metastases are the main causes of mortality, and metastatic disease as well as pathological microfractures in the tumor are common reasons for euthanasia [3,4,14].

The treatment of OSA first concerns pain management using analgesic medication (including NSAIDs, opioids, benzodiazepines, and bisphosphonates) or radiation therapy. Tumor treatment is then performed combining surgical resection (limb amputation or limb-sparing procedure) with adjuvant therapy (radiation therapy or metronomic chemotherapy) with the aim being to prevent or delay local recurrence, but also to slow down metastasis occurrence [13]. Amputation is the gold standard for the local management of primary bone tumors and, when performed alone, is associated with a median survival time of approximately 5 months [3,4]. The overall survival time is markedly increased when surgery and chemotherapy are combined, reaching a median of 1 year [3,4]. However, amputation is not always feasible due to the animal’s weakness, large size or overweight, dysplasia, previous amputation, bone metastases on other limbs, and concurrent orthopedic or neurological diseases that may preclude ambulation on the remaining three limbs. Owners’ refusal is another common and important reason to try to develop an alternative to amputation. Furthermore, even if most dogs with an amputated limb adapt to walking on three legs within a month, behavioral changes such as increased fear, aggression, or anxiety, as well as a reduction in dominance towards other dogs, have been reported in approximately one-third of them [16,17].

Over the years, advances in disease management, including limb-sparing surgery procedures, have been described. The most common limb sparing techniques consist in resecting and replacing the affected bone by a metal endoprosthesis, normal bone allograft, or ulnar autograft, thus preserving the function of the limb [1,18]. Eligibility for limb sparing includes tumors preferably located on the distal radius or ulna, involving less than 50% of the length of the bone with no extension across the joint, and requires the absence of pathological fracture. Several other parameters defined the feasibility of the limb-sparing surgery for OSA treatment. The presence of tumor metastasis, generally found in the lungs, bones, and regional lymph nodes, is a poor prognosis factor and may therefore influence the decision to perform a limb sparing surgery [7,19]. When applicable, limb function is preserved in over 80% of dogs following such limb-sparing surgeries [7]. Nevertheless, these techniques seem to have many limitations. They have been reported to be painful and are associated with tumor recurrence (in 15–25% of cases) or with complications including infections (in 30–50% of patients), resorption of the allograft, implant failure (20–40%), implant loosening, plate and allograft fracture, and allograft nonunion [7,15,20].

Calcium phosphate cements have been used since the 1980s in humans and are now considered as the gold standard procedure to support bone augmentation in many clinical situations. Calcium phosphate cements are also frequently used for bone reinforcement in fracture repair procedures and as treatment for benign bone tumors in the form of a cementoplasty [21,22,23,24,25,26,27,28,29,30]. Although cementoplasty is a minimally invasive procedure associated with a relative ease of execution, a short surgical time reducing the risk of infectious complications and a rapid postsurgical recovery, this procedure, either associated with poly-methylmethacrylate (PMMA) or calcium phosphate cement, has rarely been studied for the palliative treatment of OSA in dogs [31,32,33]. Considering its high compressive strength, PMMA cement represents an excellent material able to restore the expected bone strength. However, the exothermic polymerization of PMMA cement and the associated release of toxic residual monomers can result in osteonecrosis, which can compromise stability and increase the risk of pathological fracture [30,34]. In addition, heat production and residual toxic monomers induce the formation of fibrous tissue hampering direct contact at the bone–PMMA interface and are associated with chronic inflammation. This reaction can cause further necrosis and osteolysis of the surrounding bone [35]. In comparison, calcium phosphate cements have specific properties that make them more suitable for intra osseous injection. Indeed, they are highly biocompatible thanks to their composition close to the natural bone [36]. This biocompatibility, associated with an isothermic hardening process, avoids the activation of necrotic and inflammatory processes [37]. In addition, the calcium phosphate cement acts as a scaffold due to its porosity and provides a good osteointegration to the adjacent healthy bone tissue [38]. Finally, osteoconductivity together with bioactivity properties of the cement enhance a resorption–bone substitution process and subsequent bone remodeling.

The aim of the current non-controlled prospective study was to evaluate the effects of cementoplasty procedure with a new injectable self-hardening resorbable calcium phosphate cement (BIOCERA-VET^®^, TheraVet, Gosselies, Belgium) on primary canine appendicular OSA lesions in terms of feasibility and safety of the procedure, pain relief, bone reinforcement to prevent further pathological fractures and ensure quality of life. In this study, no particular selection was made on the stage of the disease, with the exception of the presence of pulmonary metastases and pathological fractures. Such a palliative treatment was expected to improve the animal welfare by decreasing the pain caused by the tumor and by preserving limb function even without any antitumoral activity.

## 2. Materials and Methods

### 2.1. Ethical Approval

The protocol defining all study procedures was approved by the Internal Ethics Committee of OCRvet under the protocol reference 20-CEI-06. Furthermore, the experimental nature of cementoplasty procedure, and the availability of other treatment options for this neoplasia were explained to the owners, who gave informed consent in light of this information (inform consent form provided in Supplementary File S1). In addition, only those owners who categorically refused amputation were offered the alternative therapy described in the present study. So, the enrolled animals did not suffer any “loss of chance of survival” as they would have been euthanized very quickly without this option.

### 2.2. Dogs

This multicentric, prospective, open-label, non-controlled trial involved six veterinary surgeons in France. Client-owned adult dogs (the experimental unit) of any breed, gender, or age, diagnosed with appendicular OSA were enrolled. Depending on investigational sites standard procedures, a presumptive OSA diagnosis was performed based on signalment findings, location of lesions, and radiographic or computed tomography (CT) findings, with or without associated biopsy results. Only dogs that could not undergo amputation because of various reasons including weakness, overweight, previous amputation, or owner’s refusal, were enrolled in the study. Dogs were excluded if they had pulmonary metastasis, fever, diffuse osteopenia, immunodeficiency, or poor physical condition, or if they had received chemotherapy or radiotherapy within four weeks before inclusion. The presence of an intercurrent disease interfering with the conduct of the study was also an exclusion criterion. In order to confirm the animal’s eligibility, standard hematological and biochemical analyses as well as chest radiographs were performed in addition to orthogonal X-ray views of the surgical site. Short-term analgesic drugs (non-steroidal anti-inflammatory drugs (NSAIDs), tramadol, morphine, corticosteroid), antibiotics, symptomatic medications (e.g., anti-diarrhea, anti-vomiting), and cytotoxic carboplatin-based systemic chemotherapy or metronomic oral chemotherapy (cyclophosphamide; chlorambucil), started not earlier than two weeks after surgery, were authorized throughout the study period. Once enrolled in the study, the animal’s follow-up was scheduled for 6 months. If animals met exclusion criteria during the course of the study, they were withdrawn from the study.

### 2.3. Calcium Phosphate Cement

The calcium phosphate cement (BIOCERA-VET^®^, TheraVet, Gosselies, Belgium) is a bioactive resorbable bone substitute with osteoconductive properties. The cement was provided in an easy-to-use dual-chambered syringe pre-filled with a powder (composed of 78 wt% (weight%) α-tricalcium phosphate (TCP), 10 wt% anhydrous dicalcium phosphate (DCPA) (CaHPO_4_), 10 wt% CDA, 2 wt% hydroxypropylmethylcellulose (HPMC)), and a liquid phase (5 wt% Na_2_HPO_4_ aqueous solution (liquid/powder ratio = 0.5 mL·g^−1^) and a 0.5 wt% Na_2_HPO_4_ aqueous solution (liquid/powder ratio = 0.45 mL·g^−1^)). After mixing, a final volume of 8 mL of cement was obtained. A 7G luer-lock cannula and an injection device were provided to perform the injection of the cement.

The isothermic crystallization of the cement is initiated by the reconstitution, with a setting time of 8 min and a complete hardening time of 24 h, that give rise to calcium-deficient apatite. The harden cement is characterized by a compressive strength of 13± MPa, a porosity of 63 ± 3% and a permeability of 10^−8^ m·s^−1^.

### 2.4. Percutaneous Cementoplasty Procedure

All dogs were anesthetized and monitored according to the standard procedures of the investigational sites (Supplementary File S2). When available, the injection procedure was performed under fluoroscopic guidance to aid visualization of filling of the bone defect with cement in real time. After routine preparation of the surgical site, a short skin incision was performed and a Jamshidi trocar was introduced in the tumoral cavity with an entry point in the intact cortical bone area in order to avoid additional lesions and risks of dissemination of tumoral cells. The content of the tumoral cavity was aspirated using a surgical aspirator connected to the trocar. The cannula was then introduced through the same entry point to the opposite edge of the lesion. Cement was injected in a retrograde manner, moving progressively upwards from the edge of the lesion towards the entry point, until bone filling was deemed subjectively satisfactory. In case of significant cortical lesion, cement leakage from the tumor site could be observed during the injection procedure but the amount of cement leakage was very limited. At the end of the operation, any residual cement at the entry point was washed away, the skin incision was sutured, and a soft bandage or splint was applied. An illustrated procedure is available in Supplementary File S3. The animal had to rest for 24 h until complete hardening of the product and was hospitalized for the time defined by the veterinarian.

### 2.5. Radiographic Examination

The bone cement being a radio-opaque material, orthogonal radiographical projections of the tumor site were taken immediately after surgery on D0 to check the filling of the lesion. Furthermore, similar X-rays were repeated on D28, D56, and D183 to monitor the tumoral evolution, potentially associated pathological fractures, and the postoperative aspect of the injected cement. In addition, on D56 and D183, chest radiographs were performed to detect the presence of pulmonary metastases.

### 2.6. Outcomes

Function and pain of the affected limb were evaluated in each animal just before surgery and then postoperatively using four evaluation systems.

#### 2.6.1. Veterinary Score

A veterinary score (VS) combined four dimensions: lameness, support on the affected limb, ease of lifting the contralateral limb, and pain on handling. Each dimension was assessed using a numerical rating scale ranging from 1 to 5 (Table 1). The VS is the sum of the individual scores of each dimension and ranged from 4 to 20. The VS was performed by the veterinarian at enrolment (D0, before the surgical procedure), D1, D28, D56, and D183. Veterinary Score was analyzed in terms of individual changes for each dog. The score determined at each follow-up visit for a particular dog was compared with the score of the same dog before treatment, to define the presence of an improvement or deterioration in limb function.

#### 2.6.2. 4A-Vet

The 4A-Vet composite pain scale (0–18) was completed by the veterinarian to evaluate pain intensity through the behavioral expressions and orthopedic components of pain [39]. The evaluation was performed at D0 (before the surgical procedure), D1, D28, D56, and D183. The total 4A-Vet score is the sum of the six subscale scores (overall subjective assessment, general attitude, interactive behavior, heart rate, reaction to handling of the operating area and intensity of this reaction) ranging each from 0 (no pain) to 3 (worst pain). Depending on the total score, pain was classified into 4 categories: no pain (0), mild (1 to 5), moderate (6 to 10), or severe (11 to 18).

#### 2.6.3. Canine Brief Pain Inventory

The Canine Brief Pain Inventory (CBPI) questionnaire was completed by the owner at enrolment (D0, before the surgical procedure), D28, D56, and D183 [40]. The questionnaire allows the evaluation of the severity of pain (Pain Severity Score (PSS) ranging from 0 to 10 and corresponding to the mean score of 4 items), the interference of pain with function (Pain Interference Score (PIS) ranging from 0 to 10 and corresponding to the mean score of 6 items), and the dog’s quality of life (QoL).

#### 2.6.4. Pain Visual Analog Scale

The global pain was also assessed by the owner using a Visual Analog Scale (VAS) consisting of a 100 mm-long horizontal line with vertical bars at each end and was labeled “no pain” (0) at one end and “worst pain” (100) at the other end [41]. The owner had to mark on the VAS the pain level of his dog at the moment of the evaluation. Subsequently, the score was determined by measurement of the position of the mark on the scale. The owner evaluated the animal’s global pain at enrolment (D0, before the surgical procedure), D28, D56, and D183.

### 2.7. Reporting of Analgesic and Antibiotic Consumption

The amount of prescribed analgesic and antibiotic drugs administered before and after surgery and then taken by dogs during the study period was documented.

### 2.8. Safety Evaluation

The occurrence of post-operative complications was evaluated. Major complications were defined as those potentially requiring aggressive surgical or medical therapy or patient death. Minor complications were defined as those not requiring any additional surgical intervention and could be managed conservatively [42].

### 2.9. Statistical Analysis

The dog was considered as the experimental unit. The non-parametric Wilcoxon matched-pairs signed rank test was used to compare the relative changes in the 4A-Vet, VS, PSS, PIS, QoL, and VAS scores between baseline (D0) and each time point with a significance level was set at 0.05. For the purpose of these analyses, the owners’ qualitative assessments of QoL were encoded as quantitative scores as follows: Poor = 1; Fair = 2; Good = 3; Very Good = 4; Excellent = 5. All analyses were performed by using the GraphPad Prism software package (version 8.2.1).

## 3. Results

### 3.1. Demographics

Thirteen dogs were screened/selected, one of which presented an advanced stage of the disease with severe bone lysis impeding/preventing cementoplasty as assessed by the veterinarian. Twelve large and giant-breed dogs, including five (42%) males and seven (58%) females, were enrolled in the study (Table 2).

The mean age was 7 years and 6 months (±28.47 months, range: 3–10 years and 9 months) and the mean weight was 43.71 kg (±14.89 kg, range: 30–75 kg). Tumors were mainly located at the proximal humerus (5 cases) and distal radius (4 cases). The two other locations were the proximal tibia (2 cases), and the metacarpal head bone.

Out of the 12 dogs, 2 dogs completed the 6-month follow-up period and 10 dogs were prematurely withdrawn from the study. The premature withdrawal resulted from disease progression resulting in natural death (1 case), administration of non-authorized treatment (chemotherapy or radiotherapy, 3 cases) or euthanasia at the owners’ request (5 cases). The last withdrawal resulted from a pathological fracture on the adjacent bone (ulna) of the treated bone. Nine (9) dogs were presented at the 1-month follow-up visit, six at the 2-month follow-up visit, and two at the 6-month follow-up visit (study completion).

None of the included dogs were treated with the authorized carboplatin-based systemic chemotherapy or metronomic oral chemotherapy.

### 3.2. Cementoplasty

The mean time between OSA diagnosis and cementoplasty was 20.73 (±15.70) days (minimum: 6 days; maximum: 52 days) in 11 dogs. One dog was not considered for this parameter as it had been treated once with a cementoplasty procedure 1 year earlier and had undergone a second cementoplasty procedure as part of this study due to lameness recurrence and increased bone lysis on X-rays at the time of inclusion.

The cementoplasty procedure was performed in all dogs. The median volume of bone cement injected was 10 mL (range: 4–15 mL, Table 3) depending on the size of the tumoral cavity and the level of cortical lysis. Limited bone cement leakage occurred in 2 dogs with no clinical consequence on the outcome.

**Figure 1:**
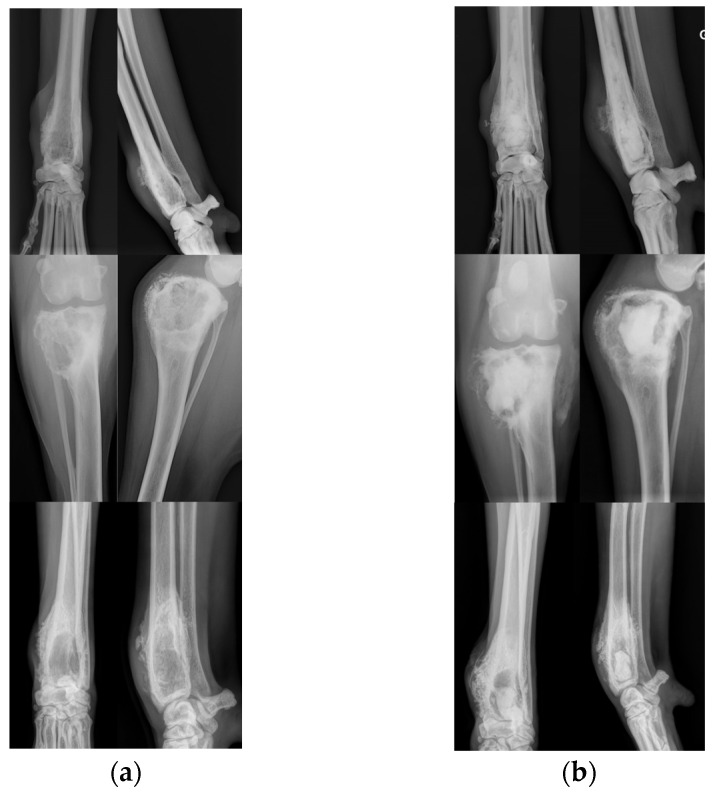
Pre- and post-operative radiographs of distal radius (dog 1 and 9) and proximal (dog 4) OSA treated by cementoplasty procedure. Cranio-caudal and latero-medial views of the cases before (**a**) and directly after (**b**) the injection of the calcium phosphate cement.

Radiographs of three cases are presented in Figure 1.

The median total surgical time for the cementoplasty procedure was 1.04 (±0.44) hours, ranging from 20 to 90 min. The hospitalization duration defined as needed by the veterinarian for the 12 dogs was between 1 and 2 days. On average, dogs were hospitalized for 48 h.

### 3.3. Veterinary Score

The veterinary score was evaluated on 10 dogs at D1 (evaluation was missing for dogs 1 and 2 and performed at D0 after the surgery for dog 5). Compared to the preoperative evaluation performed at D0, the VS decreased in 2/10 dogs on D1, 4/9 (44.44%) dogs on D28, 2/6 (33.3%) dogs on D56, and 1/2 (50.0%) dogs on D183 (Table 4).

In the nine animals that had a follow-up visit, depending on the animal and the study visit, the VS remained unchanged or increased. At their last follow-up, the VS was decreased in 5/9 (55.6%) dogs, stable in 2/9 (22.2%) animals, and increased in 2/9 (22.2%) dogs. The decrease in score varied from 14 to 36%.

### 3.4. 4A-Vet

According to the 4A-Vet score preoperative evaluation, 1 animal exhibited no pain (score = 0), 8/12 (66.7%) dogs had mild pain (score between 1 and 5), and 3/12 (25.0%) had moderate pain (score between 6 and 10) at D0 (Table 5).

The 4A-Vet score was evaluated on 10 dogs at D1 (evaluation was missing for dogs 1 and 2 and performed at D0 after the surgery for dog 5), 9 dogs at D28, 6 dogs at D56 and 2 dogs at D183. Compared to the preoperative evaluation at D0, the 4A-Vet score evaluated the day after the surgery increased in 5/10 (50.0%) dogs, decreased in 3 animals, and was stable in 2 animals. At D28, the 4A-Vet score decreased in two thirds of the animals and increased in one third compared to preoperative evaluation. At D56, an increased 4A-Vet score was observed in 2/6 (33.3%) dogs, a decreased score was reported in 3/6 (50%) animals, and a stable score was recorded in 1 (16.7%) animal compared to initial values. At study completion (D183), the two remaining animals experienced a decrease in the 4A-Vet score compared to the assessment performed before surgery. When considering the last follow-up visit for the 9 dogs that had one, 6/9 (66.7%) dogs had an improved evolution compared to D0, with a decrease in score ranging from 25 to 100%. For two dogs, a worsened evolution of the 4A-Vet score was observed compared to D0. The remaining dog had a stable evolution. Furthermore, pain remained mild throughout the study period for five animals, two animals experienced an increased pain from mild to moderate or severe, and two dogs showed a decreased level of pain within the study period.

### 3.5. Canine Brief Pain Inventory

The CBPI scores (CBPI PSS, CBPI PIS, and QoL) were evaluated on nine dogs at D28, six dogs at D56 and two dogs at D183, and nine dogs had at least one follow-up visit.

The evolution of CBPI PSS and CBPI PIS over time was similar. It was favorably improved for 5/9 dogs (55.6%) and worsened for 4/9 dogs (44.4%) between the evaluation performed at D0 and at the last follow-up visit (Table 6).

An analysis of the CBPI PSS and CBPI PIS at each visit showed both scores decreased in 6/9 (66.7%) dogs and increased in 3/9 (33.3%) dogs between the preoperative evaluation and D28. At D56, an equivalent number of dogs (3/6) experienced an increase or a decrease in CBPI PSS and CBPI PIS. At D183, however, CBPI PSS increased in 1/2 animal and decreased in the other, whereas CBPI PIS increased in the 2/2 animals remaining in the study at study termination. Interestingly, no pain and/or no pain interference with the limb function (i.e., CBPI PSS and CBPI PIS ≤ 0.5) were noticed in three dogs during their follow-up (dogs 2, 5, and 7). Finally, a QoL evaluation showed an improvement between the preoperative evaluation and the evaluation at the last follow-up visit in 56% of the cases (five out of nine animals), a stability in 22% of dogs (two out of nine animals) and a deterioration in 22% of animals (two out of nine animals). On D28, the QoL was improved for all dogs, except for two dogs whose QoL remained stable. On D56, the QoL increased for 3/6 dogs, remained stable for 1/6 dog, and decreased for 2/6 animals. At the end of the follow-up period (on D183), 1/2 animals had an improved QoL compared to D0, and the other a reduced QoL. The mean relative change in QoL scores was significantly different at D28 compared to the initial evaluation with an increase in the QoL score of 39.8% (*p* = 0.0156). The mean relative change was, however, not significant at D56 compared to D0, and no statistical analyses could be performed at D183.

### 3.6. Pain Visual Analog Scale

The pain VAS was evaluated on nine dogs at D28, six dogs at D56, and two dogs at D183, and nine dogs had at least one follow-up visit.

The pain VAS score improved in 5/9 dogs (56%) and worsened in 4/9 animals (44%) at the last follow-up visit compared to the preoperative evaluation performed at D0 (Table 7).

Remarkedly, 44% of the owners (4/9) considered that their dog no longer felt pain at their last follow-up visit. For animals with an improved score, the reduction in the VAS score ranged from 60 to 100%. In cases of a worsened score, the increase ranged from 14.3 to 27.3%. At D28, 7/9 owners (77.8%) considered that animal pain had decreased, 1/9 considered it had increased, and 1/9 considered it was stable compared to D0. At D56 and D183, pain was considered as increased or decreased by half of the owners (3/6 and 1/2, respectively). The pain had completely disappeared in 4/9 dogs at D28 with a noticeable improved evolution for one dog, whose pain decreased from 70% to 0% between D0 and D28.

The mean relative change in pain VAS score was also significantly different at D28 compared to the preoperative evaluation with a decrease in the VAS score of 55.2% (*p* = 0.0234). The difference in VAS score was not significant at D56 compared to D0.

### 3.7. Radiographic Observations

In three cases (dog 5 at D56, dog 6 at D28, and dog 7 at D28), radiographical lesion was observed, attesting disease progression and requiring the administration of chemotherapy or radiotherapy. Following the radiographic examination, euthanasia was decided for five other cases (dog 1 between D56 and D183, dog 8 between D0 and D28, dog 9 between D0 and D28, dog 10 between D0 and D28, and dog 11 between D56 and D183). The appearance of pulmonary metastasis was also checked to document the progression of the disease. No radiographically detectable thoracic metastases were identified in any of the dog on chest X-rays during the study participation.

Postoperative radiographs showed the presence of the calcium phosphate cement in the injected bone (X-rays of representative cases of injection are presented in Figure 1). The persistence of the product was observed in all dogs without any major macroscopic modification throughout the study period, demonstrating the stability of the cement inside the tumoral cavity and supporting the beneficial mechanical effects expected with the cementoplasty procedure. Radiographic evaluation also aimed to identify the occurrence of pathologic fracture. During the study follow-up, only 1 animal, with a distal radius OSA, experienced a pathological fracture. Nevertheless, the fracture did not occur at the tumor localization but on the ipsilateral ulna.

### 3.8. Analgesic and Antibiotic Consumption

All dogs, except one (dog 10), received postoperative analgesic. The durations of administration and doses varied from dog to dog, while keeping a constant dose of analgesic for each dog until the end of its follow-up. Some dogs received oral antibiotics for infection prophylaxis during their hospitalization and their return home.

Four (4) dogs (dogs 1, 2, 6, and 7) received pain killers at the time of inclusion in the study (D0) because of lameness (Supplementary File S4). On the day of surgery, all dogs received morphine, which was combined in some cases with other analgesics to limit perioperative pain. All dogs, except dog 10, received analgesics after surgery and pain treatment was prolonged after the hospitalization period in 2 cases (dogs 5 and 12) for a period of 4 to 16 postoperative days only. The analgesic treatment was maintained for all the other dogs until the end of the follow-up, except for dogs 11 and 12, which were no longer under analgesic treatment at the 28-day follow-up visit. The dosage was constant for all dogs, except one dog (dog 8) for which the dosage was decreased (Firocoxib at 7.2 mg/kg/day decreased to 5.4 mg/kg/day) 14 days after surgery.

Preventive administration (intravenous, oral or both) of antibiotics was performed for 8 dogs to prevent infection related to the surgery (Supplementary File S4). Four (4) animals (dogs 8, 10, 11, and 12) did not receive any antibiotic treatment.

### 3.9. Safety Evaluation

Ten (10) major complications were reported throughout the study period (Table 8).

All of these events were considered as being related not to the cementoplasty procedure or the bone cement, but rather to the progression of the tumoral disease (increased pain, increased bone lysis or deterioration in the general condition). They included a fracture of the operated limb (but not the operated bone) in one dog, as well as euthanasia (five dogs) or a natural death (one dog) consecutive to the deterioration of the animal’s general condition or to the progression of the disease.

Four (4) minor complications occurred during the study in four different dogs (Table 8) including edema on the operated limb 15 days after surgery, surgical site swelling immediately after surgery, surgical site infection 7 days postoperatively, and vomiting starting on the day after treatment. The development of the edema was concomitant with an overall systemic deterioration, which led to the euthanasia of the dog (dog 8). For the three other minor complications, recovery was observed within 3 to 14 days after initiation of the appropriate symptomatic treatment.

## 4. Discussion

The results of the present study provide preliminary evidence of the value of the cementoplasty procedure. Involving the injection of a new bone cement, cementoplasty represents an alternative local approach to palliative management of canine OSA in an attempt to preserve limb function, and delay or avoid amputation and further pathological fracture of the affected bone. The owners of all dogs who received this treatment had categorically refused amputation. Thus, without this option, the included dogs would have been euthanized very quickly. We can therefore reasonably assume that such palliative treatment improved the animal welfare by decreasing the pain caused by the pathology and by preserving limb function.

The profile of the dogs included in this study was consistent with OSA risk factors identified in the literature regarding large to giant breeds [5,7,8,11,12,13]. In the present study, OSAs were mainly located at the proximal humerus (5/12 dogs) and at the distal radius (4/12 dogs), with a total of 83.33% of forelimbs treated. These observations are in line with the findings made in previous studies [2,7].

The veterinary and owner tools used in this study were previously validated to evaluate post-operative pain in dogs for the 4A-Vet questionnaire [39], chronic pain for the VAS [43], severity of pain as well as pain interference with daily life and QoL for the CPBI [40,44]. Veterinary numerical scale with specific verbal description based on clinical criteria used in daily practice are also commonly used in veterinary clinical studies, in particular to assess pain and lameness, and have a good validity [45,46,47]. The use of these four measurement tools combining veterinary and owner assessments together with the comprehensive approach of pain assessment ensured the reliability of the results.

The improvement in these scores during the study period was observed in the majority of dogs. Furthermore, a consistent relationship was found in most cases between the scores resulting from the veterinary surgeons’ assessment and the scores resulting from the owners’ evaluation. The best improvement occurred generally at D28, with a tendency to decrease at D56, albeit remaining improved compared to D0. Nevertheless, the low number of animals remaining in the study at D56 made it difficult to draw clear conclusions at this time point as well as at D183. The QoL was improved for most dogs at D28 and D56. This study supports the expected effect of mechanical strength preservation on pain relief associated with a recovery of the limb function, albeit transient. The improvement in pain at D28 allowed dogs to regain some support on their limb and thus to perform routine daily activities. Nevertheless, these results showed a rapid progression of the primary tumor as no adjuvant antitumoral therapy was given during this study.

In OSA lesions, osteolytic and osteoblastic activity induce a loss of mechanical strength and stability of the concerned limb. Mechanical stress, normally without any harmful consequences, will then produce a distortion of the mechanosensitive sensory nerve fibers present in bone resulting in pain [48]. The results of this study support the expected effect of mechanical strength preservation on pain relief associated with a preservation of the limb function.

By way of palliative management of canine OSA, the cementoplasty procedure was carried out to reduce pain, reduce the risk of pathologic fractures, and improve the well-being of animals. Accordingly, no improvement in animal survival time was expected. Indeed, a low survival rate was observed in the study, with five dogs euthanized before the end of the study and one dog dying naturally. Four (4) additional dogs left the study before the end of the follow-up period. Among them, three dogs presented a tumoral progression requiring treatment by radiotherapy or chemotherapy not authorized by the protocol, and the fourth one, suffering from an OSA located on the radius, had a fracture of the ipsilateral ulna. Furthermore, the data show that the two dogs that completed the study were young (3 and 5.6 years old). In contrast, the dogs that were euthanized within a month after their inclusion were between 7.92 and 10.75 years old. This observation is consistent with studies showing that increasing age may/can have an impact on the life expectancy after treatment and that tumoral disease progression may/can be more rapid in older dogs (5).

In our study, cementoplasty procedure was performed within 1 to 2 months after OSA diagnosis. Considering that OSA is a highly progressive disease, an early detection and an early treatment after diagnosis are recommended to observe beneficial effects. Despite the absence of thoracic metastases detectable on chest X-rays, the variability of the delay between the onset of the disease and diagnosis as well as between diagnosis and surgical management may account for the high number of dogs that died before 6 months postoperatively. Early diagnosis is also fundamental to limit cortical lysis that may lead to complications such as spontaneous fractures [6,15]. In this study, the incidence of pathological fracture is, however, much lower than the incidence of 38.8% reported in the literature for dogs suffering from primary OSA [15]. Cementoplasty allows the reinforcement of the affected limb and thus to avoid pathological fractures. Among the 12 included dogs, only 1 dog experienced a spontaneous fracture. However, the fracture occurred on the ulna, which was in close contact with the primary tumor located on the radius. This fracture may have been the consequence of an increased mechanical stress on the ulna combined with local extension of the primary tumor and underlines the importance of an early detection of the disease.

Only two minor complications were evaluated as possibly related to the surgery or the product during the study (limb edema and surgical site infection). Inflammation consecutive to the surgical procedure may explain postoperative edema and surgical site infections are the most common complications after surgical procedures. The risk of surgical site infection is correlated to the operating time, so the short time required to perform the cementoplasty procedure allows surgical infection occurrence to be limited [49,50]. The variation in the operating time observed in this study is related to the variability of complexity among cases. The cementoplasty procedure could be performed in a shorter operating time than the other surgical treatment of OSA (amputation, limb-sparing) and fluoroscopic guidance, when available, can contribute to limit both tissue damages and operating time. Other expected postoperative complications such as surgical site hematoma or seroma, or cement embolism were, however, not reported throughout the study period [31].

The other complications that occurred during the study follow-up were related to OSA progression. Thus, it is noticeable that the incidence of complications reported in the present study was lower than the one observed by Böttcher et al. after percutaneous injection of PMMA in four dogs [31]. In that study, 3/4 dogs developed at least two short-term complications, and all dogs experienced long-term complications including recurrent lameness, soft tissue swelling, anemia, coughing, and acute paresis. The properties of the cement may influence the frequency of complications. Indeed, the calcium phosphate cement presents biological properties that make it particularly suitable for cementoplasty procedure. This injectable cement has a high biocompatibility with bone tissues due to its composition resulting in an excellent osteointegration. The mechanical strength of the cement obtained after the hardening process provides a rapid and stable bone support, as required in this indication. In addition, its bioactive properties allow the promotion of bone remodeling. Finally, the progressive resorbability of the cement combined with the isothermic hardening process make possible further developments for the use of the cement as a drug delivery system for specific anti-tumoral molecules in order to provide a local treatment of the tumor in addition to bone reinforcement and pain relief [38,51,52,53].

Several limitations of this study can be identified and should be acknowledged. First of all, our study provides results on a limited number of dogs and pet owners as well as veterinarians may have evaluated dogs favorably knowing they have been treated. Further research would be necessary to confirm these first observations on a larger population and with objective evaluation of limb function. Secondly, the effects of the combination of cementoplasty and adjuvant therapy on dog survival time was not evaluated. However, our results showed that early treatment of OSA using cementoplasty without any adjuvant therapies led to an improvement in both limb function and quality of life in dogs with various presentations of osteosarcoma. Finally, cement filling is facilitated under fluoroscopic control, which allows the real-time monitoring of the procedure. If fluoroscopy was not available, a radiographic control was performed at the end of the procedure but did not allow the surgeon to adjust to optimal filling. Nevertheless, the results of the present study showed that beneficial effects were obtained after cement injection, despite various presentations and locations of the primary tumor.

## 5. Conclusions

The cementoplasty procedure, involving the injection of a new calcium phosphate bone cement, is a feasible and safe palliative treatment when amputation is not possible or refused by the owners. This minimally invasive surgical procedure is beneficial for limb preservation, pathological fracture prevention, and pain relief effects even if limited to the short term in this study. As expected, considering the palliative nature of the treatment, no effect on tumoral disease progression and the related survival time was observed.

This study provides the first evidence of the feasibility, safety. Further studies are needed to obtain data on a larger number of patients, and also to determine whether the cementoplasty procedure could have an impact on disease progression.

## Figures and Tables

**Table 1:** Veterinary Score.

Clinical Signs	Score	Description
Lameness	1	Stands and walks normally
	2	Stands normally and walks with a slight limp
	3	Stands normally and walks with a severe limp
	4	Stands abnormally and walks with a severe limp
	5	Does not stand up easily and walks with a severe limp
Support on the affected limb	1	Normal support when standing and walking
	2	Normal support when standing but limping when walking
	3	Partial support when standing and walking
	4	Normal support when standing but no support when walking
	5	No support when standing and walking
Ease of lifting the contralateral limb	1	Can lift the contralateral limb without problems and has normal support
	2	Accepts with minimal resistance to lift the contralateral limb and has normal support
	3	Accepts with moderate resistance to lift the contralateral limb
	4	Significant resistance to lift the contralateral limb
	5	Refuses to lift the contralateral limb
Pain on handling	1	No pain on limb manipulation
	2	Minimal pain on limb manipulation
	3	Moderate pain on limb manipulation
	4	Severe pain on limb manipulation
	5	Limb cannot be manipulated

**Table 2:** Characteristics of the studied dog population and overview of clinical details.

Subject	Breed	Breed Size	Sex	Age at Inclusion	Weight (kg)	OSA Location
1	Akita Inu	Large	Female	5 years 1 month	32	Distal radius
2	Great Dane	Giant	Male	7 years 11 months	75	Distal radius
3	Labrador Retriever	Large	Female	5 years 7 months	30	Proximal humerus
4	Newfoundland	Giant	Female	3 years	70	Proximal tibia
5	German Shepherd	Large	Female	9 years 3 months	32	Proximal metacarpus
6	White Swiss Shepherd	Large	Female	10 years 2 months	33.5	Proximal humerus
7	Dogue de Bordeaux	Giant	Female	5 years 7 months	47	Proximal humerus
8	Bullmastiff	Giant	Male	10 years 9 months	31.5	Proximal humerus
9	Pyrenean Mountain Dog	Giant	Female	7 years 11 months	39	Distal radius
10	Old English Sheepdog	Large	Male	9 years 9 months	42.5	Proximal humerus
11	Golden Retriever	Large	Male	6 years 5 months	44	Proximal tibia
12	Rottweiler	Large	Male	8 years 7 months	48	Distal radius

**Table 3:** Overview of animals’ treatment.

Subject	Volume Injected (mL)	Operation Time (h)	Duration of Hospitalization (Days)
1	8	1.5	2
2	15	1.2	1
3	12	1.25	1
4	5	1.5	2
5	5	0.75	1
6	13	1.5	2
7	13	1.5	2
8	10	0.5	2
9	13	1	2
10	7	0.3	2
11	4	0.5	2
12	10	1	2

**Table 4:** Evolution of the veterinary score per animal over time.

Subject	D0	D1	D28	D56	D183
1	7	6	7	7	NA
2	9	NA	6	7	NA
3	11	NA	12	12	12
4	7	8	9	10	5
5	4	4	3	3	NA
6	14	14	12	NA	NA
7	11	13	7	NA	NA
8	17	15	NA	NA	NA
9	16	16	NA	NA	NA
10	9	11	NA	NA	NA
11	6	6	7	11	NA
12	12	12	12	NA	NA

NA: Not available due to withdrawal of the animal from the study or assessment not performed.

**Table 5:** Evolution of the 4A-Vet score per animal over time.

Subject	D0	D1	D28	D56	D183
1	2	3	4	2	NA
2	5	NA	1	3	NA
3	8	NA	4	3	6
4	2	1	1	6	1
5	5	3	0	0	NA
6	9	8	12	NA	NA
7	4	6	1	NA	NA
8	5	6	NA	NA	NA
9	0	2	NA	NA	NA
10	4	7	NA	NA	NA
11	2	2	3	8	NA
12	10	10	5	NA	NA

NA: Not available due to withdrawal of the animal from the study or assessment not performed.

**Table 6:** Evolution of the CBPI PSS, PIS, and QoL per animal over time.

Subject	Evaluation	D0	D28	D56	D183
1	PSS Score	4.25	5.5	4.75	NA
	PIS Score	2.83	7.17	5.50	NA
	QoL	Fair	Good	Very good	NA
2	PSS Score	3.25	0	0	NA
	PIS Score	3.20	0	0	NA
	QoL	Very good	Excellent	Excellent	NA
3	PSS Score	6.25	3.75	1.75	7.75
	PIS Score	3.50	3.67	1.17	8
	QoL	Good	Very good	Very good	Poor
4	PSS Score	3.75	4.75	6.25	2.5
	PIS Score	0.50	8.50	6.83	4.83
	QoL	Very good	Very good	Good	Excellent
5	PSS Score	2.25	0	0.5	NA
	PIS Score	6.83	0	0.33	NA
	QoL	Good	Excellent	Good	NA
6	PSS Score	4.75	7	NA	NA
	PIS Score	5.67	5.17	NA	NA
	QoL	Fair	Fair	NA	NA
7	PSS Score	3	0	NA	NA
	PIS Score	6	0.33	NA	NA
	QoL	Fair	Very good	NA	NA
8	PSS Score	5.25	NA	NA	NA
	PIS Score	6.67	NA	NA	NA
	QoL	Fair	NA	NA	NA
9	PSS Score	1.5	NA	NA	NA
	PIS Score	7.5	NA	NA	NA
	QoL	Very good	NA	NA	NA
10	PSS Score	6.5	NA	NA	NA
	PIS Score	6	NA	NA	NA
	QoL	Fair	NA	NA	NA
11	PSS Score	5.75	1.75	7.25	NA
	PIS Score	3.33	1.67	9.67	NA
	QoL	Good	Very good	Poor	NA
12	PSS Score	5.25	0	NA	NA
	PIS Score	7.67	4.33	NA	NA
	QoL	Fair	Good	NA	NA

NA: Not available due to withdrawal of the animal from the study or assessment not performed.

**Table 7:** Evolution of the VAS per animal over time.

Subject	D0	D28	D56	D183
1	40	40	50	NA
2	20	0	0	NA
3	65	30	25	80
4	50	40	80	20
5	40	0	0	NA
6	55	70	NA	NA
7	30	0	NA	NA
8	65	NA	NA	NA
9	10	NA	NA	NA
10	80	NA	NA	NA
11	70	35	80	NA
12	70	0	NA	NA

NA: Not available due to withdrawal of the animal from the study or assessment not performed.

**Table 8:** Overview of complications and final status at the end of study participation.

Subject	Minor Complications	Major Complications	Reason for Withdrawal	End of the Study Participation
1	No	Increase in pain at the tumoral site	Euthanasia	Before D183
2	No	Natural death	Death	Before D183
3	No	No	NA	D183 (completion of the study)
4	No	No	NA	D183 (completion of the study)
5	No	Bone metastasis on another limb requiring start of chemotherapy	Required chemotherapy	Before D183
6	No	Deterioration in general condition requiring start of radiotherapy treatment	Required radiotherapy	Before D56
7	No	Deterioration in general condition requiring start of radiotherapy treatment	Required radiotherapy	Before D56
8	Generalized edema	Deterioration in general condition	Euthanasia	Before D28
9	Skin swelling at injection site	Deterioration in general condition	Euthanasia	Before D28
10	No	Increased pain at the tumor site	Euthanasia	Before D28
11	Surgical site infection	Increased bone lysis at the tumor site	Euthanasia	Before D183
12	Vomiting	Fracture of another limb	Fracture of ulna	Before D56

## Data Availability

The original contributions presented in the study are included in the article/Supplementary Material, further inquiries can be directed to the corresponding author.

## Supplementary Materials

The following supporting information can be downloaded at: https://www.mdpi.com/article/10.3390/ani14101460/s1, Supplementary File S1: owner’s informed consent; Supplementary File S2: anesthetic treatments; Supplementary File S3: illustrated cementoplasty procedure; Supplementary File S4: analgesic and antibiotic treatments.
