# Supplementary material for: Pilot Clinical Trial to Evaluate In Situ Calcium Phosphate Cement Injection for Conservative Surgical Management of Appendicular Osteosarcoma in Dogs
Source: Animals (Basel). 2024 May 14;14(10):1460. doi: 10.3390/ani14101460 (PMC11117317; doi:10.3390/ani14101460)
Supplement: Supplementary file 1 [file animals-14-01460-s001.zip › Molle et al, Cementoplastie for palliative treatment of osteosarcoma in dog_Supplementary file S1.pdf]

CONFIDENTIAL

<u>1st part: INFORMATION FOR OWNERS</u>

Dear Madam, dear Sir,

Your animal is eligible to participate in a clinical field study conducted by TheraVet (the Sponsor of the study located in Jumet, Belgium). The goal of this study is to evaluate the efficacy and safety of a bone cement, in the treatment of osteosarcomas or in case of bone surgery, on the preservation of function of the affected limb.

According to the current regulation, a written confirmation of your agreement is necessary for the inclusion of your animal in the study.

Your participation is completely free and voluntary. Before any decision, take the time to read this information sheet and ask questions. Whatever you decide (participate or not in the study), your animal will continue to receive all the attention and care of your veterinarian.

Once your veterinarian has determined that your animal has met all of the criteria for enrollment, your animal can be included in the study. Below are the key points you should know about participating in the study.

<u>Treatments:</u> The product is a synthetic injectable bone void filler cement, obtained after reconstitution of a calcium phosphate powder with a liquid.

<u>Length of study:</u> If your animal is enrolled in the study you can expect to participate for a period of:

- 6 months if your animal is suffering from osteosarcoma;
- 8 weeks if your animal is undergoing bone surgery.

Once your animal starts the study, treatment will be given once and then your animal will be followed for the period corresponding to his pathology.

<u>Number of visits:</u> You will have to bring your animal back to the clinic/hospital at:

- **Visit 0 – day 0** for the injection of the product. Your dog may be hospitalized one day after administration of the product, if your Veterinarian considers it necessary. This period of hospitalization may be extended.
- **Visit 1 – day 28** (Week 4) +/- 7 days, then **Visit 2 – day 56** (Week 8) +/- 7 days, and **Visit 3** – **day 183** (Month 6) +/- 14 days after inclusion visit, if your animal is suffering from <u>osteosarcoma</u>
- **Visit 1 – day 56** (Week 8) +/- 7 days if your animal is undergoing <u>bone surgery</u>.

The timing of the visits is important. If you know today that you will not be able to make these visits on the scheduled dates, you should discuss this with your veterinarian before starting the study.

<u>Risks associated to medical device or study procedures</u>:

During surgery, the use of general anesthesia is essential. However, this anesthesia can induce adverse events such as hypotension (the most common adverse event). With the exception of the pain and quality of life assessment that you will perform, the other examinations performed during the follow-up period are the standard examinations of patients undergoing bone surgery or being treated for osteosarcoma.

Postoperative complications may occur in each indication, including:
- Bone complications, mainly related to the disease:
    - Occurrence of spontaneous fracture in animals with osteosarcoma;

- Non-consolidation of the bone for animals having undergone bone surgery.

- Wound complications mainly related to surgical act, such as hematomas, seroma and infection.

These complications are not directly related to the medical device and may occur with standard treatment.

Study procedures:

The following exams will be performed during the study in order to evaluate the efficacy of the study treatment and to follow the general health of your animal:

- **Inclusion visit (D-10 to D0):**
  - Clinical examination including weighing and temperature measurement;
  - Blood test;
  - X-ray of the surgical site;
  - Chest X-ray only for the treatment of osteosarcomas;
  - Evaluation of function and pain by the veterinarian;
  - Evaluation of pain and quality of life by yourself.
- **Visit 0 (D0):**
  - General anesthesia for injection;
  - Injection of bone cement;
  - X-ray of the surgical site.
- **Hospitalization (D0 to D1)**
  - Evaluation of function and pain by the Veterinarian.
- **For each follow-up visit, the following examinations will be carried out**
  - Clinical examination including weighing and temperature measurement;
  - X-ray of the surgical site;
  - Evaluation of function and pain by the Veterinarian;
  - Evaluation of pain and quality of life by yourself;
  - Chest X-ray, only for V2 and V3 of animals included for osteosarcoma.

Your responsibilities: You will need to do the activities listed below throughout the study. If you are unsure about whether you can meet these requirements, please discuss this with your veterinarian now.

1. Record information about your animal's pain.
2. Record information about anything abnormal or unusual with your animal.
3. Recordings are to be made by the same individual, as much as possible.
4. Bring your animal at each visit at the scheduled dates and times.
5. Tell your veterinarian promptly if your animal has any medical emergency or if you decide to withdraw your animal from the study.

Possible benefit of the study for your pet: The efficacy and safety of this medical device has already been demonstrated in humans. It is currently used in the treatment of human bone fractures. However, it is not yet available on the veterinary market. This medical device is intended to preserve the function of the affected limb by reducing your pet's pain, improving bone consolidation and reducing the occurrence of spontaneous fractures in animals with osteosarcoma. We hope to observe this beneficial evolution in your animal although this cannot be guaranteed.

Stopping or withdrawing from the study: You and your veterinarian will have the option to withdraw your animal from the study at any time. Also, the Sponsor reserves the right to stop the study at any time, and to exclude individual animals from the study.

<u>Confidentiality by the Owner:</u> You must not disclose the study details, the study treatment, or name of Sponsor to any third parties not involved in the study, including through any social media or blogs.

<u>Data protection</u>: All medical data from your animal linked to the study, as well as your personal data (first name and last name only), as far as these data are necessary for the study, will be collected in a computer file. That will allow an automated process of all data generated in this study to analyze the results of the study.

Your identity and those from your animal will be kept confidential by the Sponsor (the Treatment responsible). The results of this study may be published in a medical journal and/or presented to the authorities in charge of evaluation of the product before it is marketed.. Under no circumstances will your identity or the name of your pet be revealed.

Data collected from your animal and your identity will be shared with other companies located in France which have been mandated by the Sponsor respectively, 1) to verify the quality of the study data collected by the veterinarian (the Study Monitor: OCR – 59120 Loos), and 2) processing them in order to analyse the results of the study (The Data Processor: Soladis Digital – 59100 Roubaix). They will protect the data in accordance with the requirements of the French law on the personal data protection.
The study data will be able also to be sent to the French or other regulatory authorities, and to other organisations working in collaboration with the Sponsor in Europe.

Your consent to take part in this study therefore also implies your consent to the use of your personal data for the purposes described in this information sheet and to their transmission to the aforementioned people and authorities.

In accordance with the Regulation (EU) 2016/679, known as the General Data Protection Regulation (“GDPR”) and Law No. 78-17 of 6 January 1978 on to computers, files and freedoms (known as "Informatique et Libertés"), as amended by the law n° 2018-493 of June 20, 2018 relative to the protection of personal data, you have the right to access, rectify or delete your data, the right to limit their processing and object to the transmission of your data by withdrawing your consent at any time without having to justify it as well as the right to your data portability. If you want to exercise your rights or have any questions relating to how your data are being processed, you may contact your veterinarian using contact details indicated in the last page of this document.

Finally, if you have a complaint concerning the processing of your data, you can contact the Data Protection Authority who ensures that privacy is respected in France when personal data are processed:
Commission Nationale de l’Informatique et des Libertés (CNIL)
3 Place de Fontenoy – TSA 80715 – 75334 Paris Cedex 07.

<u>Financial implications</u>: The Sponsor will pay for the visits defined by the protocol, the examinations (bone and chest X-rays), the blood sample collected during the inclusion visit and the associated analyses. The study medical device will be provided free of charge.
On the other hand, the costs inherent to the surgery, the various follow-up consultations outside the protocol, and the treatments prescribed during and after the surgery will remain at your expense.

2nd part: OWNER'S CONSENT

This consent form must be signed by the Owner, thereby authorizing enrollment of his/her animal(s) into the study, before any study procedure may be performed.

**Animal's Name :**		**Case no. :** |__||__| . |__| |__|
**Owner's Name :** *capital letters* **:**	**First :**	**Last :**
**Investigator's Name:**		
**Phone :**	|__||__| . |__| |__| . |__||__| . |__| |__| . |__| |__| in case of an emergency	

1. I hereby certify that I am the Owner (or fully authorized representative) of the above-named animal, and that I am at least 18 years of age.
2. I have read and fully understood the Information on pages 1 to 3 of this document. The Investigator (the veterinarian) has explained all aspects and risks of the study. The Investigator has satisfactorily answered all my questions. I have been fully informed of and understand the risks associated to the study treatment and procedures.
3. I voluntarily allow my animal to participate in this study. I understand that I am free to withdraw my animal from the study at any time. I will inform the Investigator immediately if I decide to withdraw my animal from the study.
4. I will inform the Investigator of all medication that my animal has received prior to the study period to the best of my knowledge.
5. During the study, I will only treat my pet with the treatments prescribed or approved by the Investigator.
6. During this study, I will immediately inform the Investigator of any unusual or unexpected occurrences.
7. I will bring my animal to the scheduled visits at the clinic/hospital. I will inform the clinic/hospital as early as possible if I am unable to bring my animal on any of the scheduled visits.
8. I understand that data collected during the study will be used to determine the safety and efficacy of this investigational medication and may be reviewed by the relevant authorities
9. I agree to not hold TheraVet, the veterinary clinic/hospital/practice or the Investigator liable in the event that my animal would have an adverse reaction during the course of this study.
10. I understand and hereby agree that my or my pet's personal data may be processed and used for the purposes of this study. I accept the computerized processing of the data collected as part of the study in accordance with Law No. 78-17 of 6 January 1978 as amended, and European Regulation (EU) 2016/679. I could exercise my rights of access, rectification, deletion, limitation of data treatment, my right to object at any time to the processing of my or my pet's personal data, directly to the veterinarian who is following my animal in the study and my right to data portability.
11. I will not disclose information regarding study details to any third parties not involved in the study.

In duplicate, one being for me:

Owner signature: ________________________ **Date:** ____/____/________
(or fully authorized representative) dd mm yyyy

Investigator signature: ________________________ **Date:** ____/____/________
dd mm yyyy
