# Supplementary material for: Pilot Clinical Trial to Evaluate In Situ Calcium Phosphate Cement Injection for Conservative Surgical Management of Appendicular Osteosarcoma in Dogs
Source: Animals (Basel). 2024 May 14;14(10):1460. doi: 10.3390/ani14101460 (PMC11117317; doi:10.3390/ani14101460)
Supplement: Supplementary file 1 [file animals-14-01460-s001.zip › Molle et al, Cementoplastie for palliative treatment of osteosarcoma in dog_Supplementary file S3.pdf]

Illustrated percutaneous cementoplasty procedure

Step 1 - Short skin incision

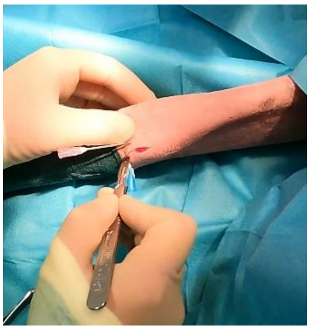

Step 2 - Jamshidi trocar introduction

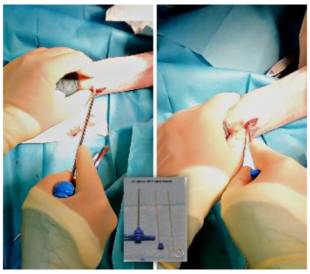

Step 3 - Aspiration of the bone cavity

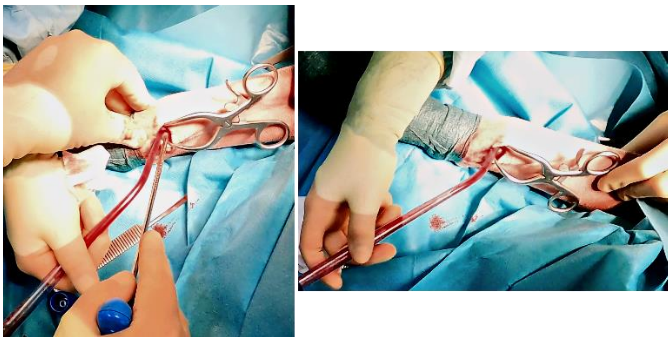

Step 4 - Introduction of the cannula for injection

Insert the cannula via the access point into the bone defect. The end of the cannula should be placed at the opposite edge of the lesion and confirm its position with diagnostic imaging. A slow injection is recommended in order to limit the pressure within the syringe and facilitate the outflow of the product. Change the angulation of the cannula and keep filling in multiple directions. Always confirm with your chosen imaging modality.

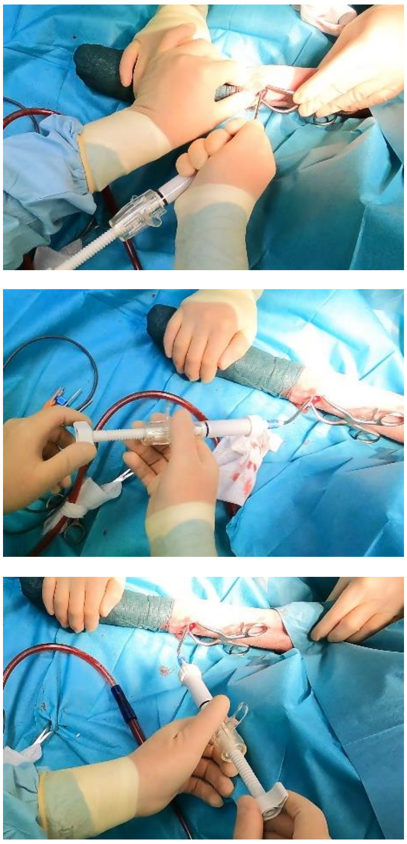

Step 5 - Filling of the cavity

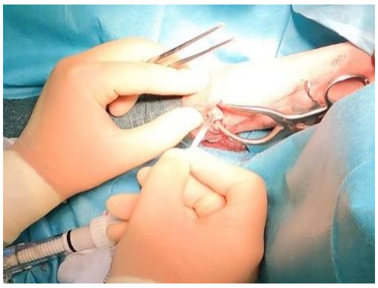

Filling a minimum of 50 % of the cavity adjacent to the still intact bone is highly recommended. It may be required to approach the lesion orthogonally to achieve appropriate debridement and subsequent filling. When cement starts to overflow out of the access point check with the imaging technique the filling situation, this is a sign that the available space has been properly filled. Exit the access point and fill it with cement, until it is flush with the adjacent cortical bone.
Finally, clean the exit area of any possible residual product.
